# A novel *in vitro* model of the small intestinal epithelium in co-culture with ‘gut-like’ dendritic cells

**DOI:** 10.1093/discim/kyad018

**Published:** 2023-10-07

**Authors:** Luke J Johnston, Liam Barningham, Eric L Campbell, Vuk Cerovic, Carrie A Duckworth, Lisa Luu, Jonathan Wastling, Hayley Derricott, Janine L Coombes

**Affiliations:** Department of Infection Biology, Institute of Infection and Global Health & School of Veterinary Science, Faculty of Health and Life Sciences, University of Liverpool, Liverpool, UK; Wellcome-Wolfson Institute for Experimental Medicine, School of Medicine, Dentistry and Biomedical Sciences, Queen’s University Belfast, Belfast, UK; Department of Infection Biology, Institute of Infection and Global Health & School of Veterinary Science, Faculty of Health and Life Sciences, University of Liverpool, Liverpool, UK; Wellcome-Wolfson Institute for Experimental Medicine, School of Medicine, Dentistry and Biomedical Sciences, Queen’s University Belfast, Belfast, UK; Institute of Molecular Medicine, RWTH University Hospital, Aachen, Germany; Institute of Systems, Molecular and Integrative Biology, Faculty of Health and Life Sciences, University of Liverpool, Liverpool, UK; Department of Infection Biology, Institute of Infection and Global Health & School of Veterinary Science, Faculty of Health and Life Sciences, University of Liverpool, Liverpool, UK; Department of Infection Biology, Institute of Infection and Global Health & School of Veterinary Science, Faculty of Health and Life Sciences, University of Liverpool, Liverpool, UK; College of Health, Medicine and Life Sciences, Brunel University London, Kingston Lane, Uxbridge, Middlesex, UK; Department of Infection Biology, Institute of Infection and Global Health & School of Veterinary Science, Faculty of Health and Life Sciences, University of Liverpool, Liverpool, UK; Department of Infection Biology, Institute of Infection and Global Health & School of Veterinary Science, Faculty of Health and Life Sciences, University of Liverpool, Liverpool, UK; School of Pharmacy and Life Sciences, Robert Gordon University, Aberdeen, UK

**Keywords:** dendritic cell, epithelial cell, mucosal immunology, enteroid, flow cytometry

## Abstract

Cross-talk between dendritic cells (DCs) and the intestinal epithelium is important in the decision to mount a protective immune response to a pathogen or to regulate potentially damaging responses to food antigens and the microbiota. Failures in this decision-making process contribute to the development of intestinal inflammation, making the molecular signals that pass between DCs and intestinal epithelial cells potential therapeutic targets. Until now, *in vitro* models with sufficient complexity to understand these interactions have been lacking. Here, we outline the development of a co-culture model of *in vitro* differentiated ‘gut-like’ DCs with small intestinal organoids (enteroids). Sequential exposure of murine bone marrow progenitors to Flt3L, granulocyte macrophage colony-stimulating factor (GM-CSF) and all-*trans*-retinoic acid (RA) resulted in the generation of a distinct population of conventional DCs expressing CD11b^+^SIRPα^+^CD103^+/−^ (cDC2) exhibiting retinaldehyde dehydrogenase (RALDH) activity. These ‘gut-like’ DCs extended transepithelial dendrites across the intact epithelium of enteroids. ‘Gut-like’ DC in co-culture with enteroids can be utilized to define how epithelial cells and cDCs communicate in the intestine under a variety of different physiological conditions, including exposure to different nutrients, natural products, components of the microbiota, or pathogens. Surprisingly, we found that co-culture with enteroids resulted in a loss of RALDH activity in ‘gut-like’ DCs. Continued provision of GM-CSF and RA during co-culture was required to oppose putative negative signals from the enteroid epithelium. Our data contribute to a growing understanding of how intestinal cDCs assess environmental conditions to ensure appropriate activation of the immune response.

## Graphical Abstract

**Graphical Abstract:**
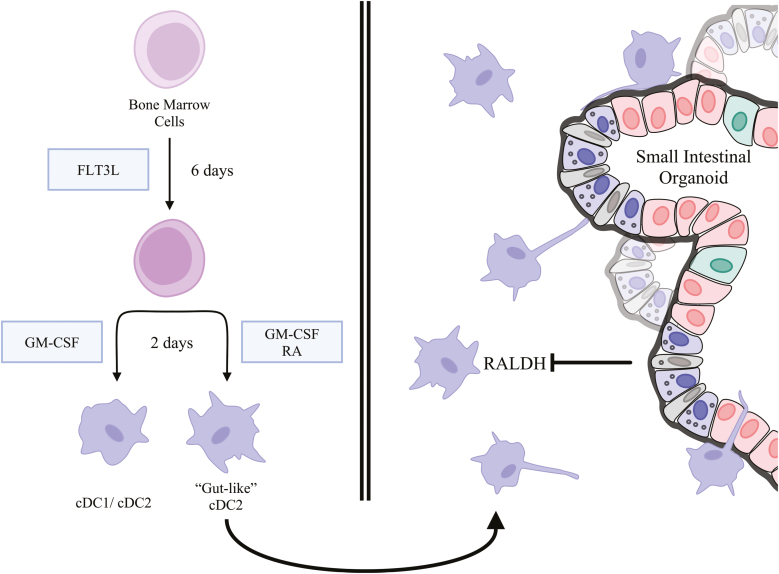

## Introduction

Conventional dendritic cells (cDCs) in the intestine are multi-functional: they coordinate protective immunity to pathogens, dampen potentially damaging immune responses to the microbiota or dietary antigens, and mediate tissue repair. However, much remains to be discovered about the signals that cDCs receive in the intestine that allow them to accurately assess local conditions and act as custodians of intestinal homeostasis. Central to this decision-making process are likely to be bi-directional interactions between the intestinal epithelium and closely associated cDCs. Small intestinal organoids (enteroids) offer a tractable tool to investigate the molecular mechanisms underpinning cDC–epithelium interactions [1–4].

Bone marrow precursors differentiate into pre-mucosal DCs whose development is regulated by all-*trans*-retinoic acid (RA). RA also imprints the gut-homing receptor α4β7 on pre-mucosal DCs that home preferentially to the intestines to replenish CD103+ cDC pools [5]. In the lamina propria (LP) of the intestine, cDCs comprise both cDC1 (CD103^+^CD11b^−^) and cDC2 (CD103^−^CD11b^+^ or CD103^+^CD11b^+^) subsets. Flt3L is required for cDC development via a pre-cDC progenitor in the bone marrow. While cDC1 dominate in the colonic LP, cDC2 are the dominant subset in the small intestinal LP (siLP), reflecting high local concentrations of RA and TGF-β, which bias differentiation of precursors towards the cDC2 lineage [6–9]. Dietary vitamin A is converted to RA by intestinal epithelial and stromal cells through the action of retinaldehyde dehydrogenases (RALDH). Segmented filamentous bacteria also produce RA from dietary vitamin A that promotes epithelial defence against infection [10]. RA initiates a transcriptional programme that produces characteristic features of intestinal cDCs, including expression of an aldehyde dehydrogenase, *Aldh1a2*. This allows intestinal cDCs to produce their own RA, which they use to induce Treg differentiation and gut tropism in lymphocytes. The cDC2 population is dramatically reduced in mice fed a vitamin A-deficient diet [8]. Maintenance of cDC2 in the intestine also depends on granulocyte macrophage colony-stimulating factor (GM-CSF) derived from stromal cells or innate lymphoid cells in the LP [11–14]. Thus, Flt3L, GM-CSF, and RA act together to regulate homeostasis and function of the cDC2 population in the intestine.

While RALDH activity in intestinal cDCs is central to their ability to regulate adaptive immune responses, much remains to be understood about how RALDH activity is regulated [15–17]. Notably, RALDH activity in intestinal cDCs is not universal, varying by location, cDC subset, and inflammatory state [6, 7, 18–20]. Various factors have been suggested to regulate RALDH activity in intestinal cDCs, including GM-CSF, dietary glucose, IL-4/13, TLR ligands, β1 integrins, PGE2, Galectin-9, and RA itself [13, 21–26]. How these signals are integrated in homeostasis or infection to appropriately regulate RALDH activity in cDC, and how they become dysregulated in chronic inflammation, remains unclear.

cDCs sample antigen in the intestine through a variety of routes. This includes the projection of *trans*-epithelial dendrites (TED) into the luminal space, although it remains unclear whether this is primarily a property of bona fide cDC or CX3CR1^+^ mononuclear phagocytes. Projection of TED is influenced by CX3CR1 signalling, TLR ligands, and lactate [27–29]. However, this method of antigen sampling is difficult to study *in vivo* where surgical preparation of the intestine for imaging may itself influence TED formation. Intestinal DCs may also migrate into the epithelial cell layer from the LP, resulting in an altered function state [3, 28].

Enteroid cultures present an opportunity to carefully dissect how interactions between cDCs and intestinal epithelial cells regulate RALDH activity and extension of TED. Here, we describe the optimization of a tractable model system where cDC2 with a gut-like phenotype are differentiated from unsorted bone marrow progenitors and co-cultured with enteroids. We provide evidence that gut-like cDCs extend TED, and show that continuous exposure to GM-CSF/RA is required to protect DCs from epithelial-derived signals that oppose RALDH activity. Our data contribute to a growing understanding of how intestinal cDCs assess environmental conditions to ensure appropriate activation of the immune response.

## Materials and methods

### Mice

Small intestines were harvested from female-specific pathogen-free C57B1/6J mice aged between 6 and 12 weeks (Charles River, Margate, UK) and, in some experiments, from mT/mG mice (Gt(ROSA)26Sor^tm4(ACTB-tdTomato,-EGFP)Luo^, The Jackson Laboratory). Same-sex litter mates were housed together in individually ventilated cages with up to five mice per cage. All mice were kept under a lighting cycle of 12 h:12 h light:dark, with *ad libitum* access to food and water. Nesting material, shelter, and polyvinyl chloride (PVC) piping were included for environmental enrichment. Mice were housed under pathogen-free conditions in the Biomedical Services Facility of the University of Liverpool, accredited by the UK Home Office and operating under UK legislation Animals Scientific Procedures Act (ASPA) 1986. All procedures were performed on adult female mice aged 8–12 weeks under project license_7008747, conforming to the guidelines from Directive 2010/63/EU of the European Parliament on the protection of animals used for scientific purposes.

Bone marrow cells were isolated from the femurs and tibias of female C57B1/6J mice only. Mice were culled by cervical dislocation as outlined in Schedule 1 of the ASPA 1986. Tissue harvest protocols were reviewed and approved by the University of Liverpool Animal Welfare and Ethical Review Board.

### Murine enteroid culture

Enteroid cultures were generated as described previously [30]. Murine jejunal tissues were harvested by dissection and any fat tissue was removed. Tissues were sliced longitudinally to expose luminal contents, washed three times in phosphate buffered saline (PBS), and cut into ~0.5 cm sections. Sections were incubated in ethylenediamine tetraacetic acid (EDTA) (30 mM in PBS) (Corning, Loughborough, UK) for 5-min cycles and subsequent vigorous shaking for 15 s in PBS dissociated crypt units. Fractions from each cycle were assessed by microscopy and those containing intact crypts largely free from contaminating villi were passed through a 70-μm strainer to remove villi. Isolated crypts were counted, centrifuged at 300×*g* for 5 min at 4°C and re-suspended at 10 crypts/μl in 70% Growth Factor Reduced, Phenol Red Free, Matrigel^®^ (Corning) and 30% IntestiCult OGM Mouse Basal Medium (StemCell, Cambridge, UK) and plated. The Matrigel^®^ cultures were polymerized at 37°C in 5% v/v atmospheric CO_2_ for 20 min, and overlaid with IntestiCult OGM Mouse Basal Medium. Enteroid medium was replaced every 3–4 days and cultures were split on days 7 or 8 at a ratio of 1:3 or 1:4.

### Bone marrow isolation and culture into intestinal DCs

Femurs and tibias of mice were removed by dissection and attached muscle were removed. Bones were soaked in 70% ethanol for 5 min. The epiphyses were cut using a scalpel and the bone marrow was flushed out using a syringe and needle with basal culture medium (RPMI 1640 supplemented with 10% v/v fetal bovine serum (FBS), 50 μM 2-mercaptoethanol, 2 mM L-glutamine, and 1% v/v Penicillin/Streptomycin). Cells were centrifuged at 300×*g* for 5 min and re-suspended in ACK lysis buffer for 3 min at room temperature. Cells were passed through a 70 μm filter, centrifuged and re-suspended in basal culture medium and factors were added as indicated in results. The factors used to optimize differentiation of bone marrow cells into dendritic cells (DCs) with an intestinal phenotype were 200 ng/ml recombinant FMS-like tyrosine kinase 3 ligand (Flt3L, Biolegend), 20 ng/ml recombinant GM-CSF (Biolegend), and 1 μM all-*trans*-Retinoic acid (RA, Sigma). Cells were cultured at 37°C, 5% v/v atmospheric CO_2_ for 8 days before purification for CD11c^+^ cells by positive selection using MACS LS columns (Miltenyi) according to the manufacturer’s protocol.

### Isolation of dendritic cells from the siLP

The small intestines of mice were harvested with fat and Peyer’s Patches subsequently were removed. Tissues were cut open longitudinally and washed in PBS three times and cut into approximately 0.5 cm sections. Epithelial cells were removed by incubations in Hanks’ balanced salt solution (HBSS) supplemented with 5% v/v FBS, 5 mM EDTA, and 10 mM HEPES buffer solution in an orbital shaker for 20 min, 250 rpm at 37°C. This was repeated for a total of three times with vigorous shaking in PBS between incubations. The tissues were minced using scissors and incubated in HBSS with 1.5 mg/ml collagenase VIII (Sigma) and 40 μg/ml DNAse I (Sigma) for 20 min in an orbital shaker at 200 rpm, 37°C followed by 20 s on a vortex for thorough dissociation. The suspension was passed through 70 μm and 30 μm filters. Cells were enriched for CD11c^+^ by positive selection with CD11c microbeads (Miltenyi) through two LS columns (Miltenyi) as per the manufacturer’s instructions.

### Antibody staining of DCs and flow cytometry analysis

Before staining for cell surface markers, DCs were stained for the intracellular enzyme aldehyde dehydrogenase (RALDH) using the ALDEFLUOR kit (StemCell, Cambridge, United Kingdom) in ALDEFLUOR buffer, as per the manufacturer’s instructions with some alterations. Final concentrations of ALDEFLUOR reagent and DEAB inhibitor control were 365 nM and 90 μM, respectively. Samples were incubated for 30 min at 37°C and washed with ALDEFLUOR buffer for subsequent cell surface staining.

Cells were first blocked against the CD16/32 receptor in buffer (PBS with 2% v/v FBS or ALDEFLUOR buffer) on ice for 30 min. Fluorescently conjugated antibodies were added to cell samples and incubated on ice, in the dark, for 30 min. The following antibodies were acquired from Biolegend and used for staining: I-A/I-E (MHCII)-BV421 (M5/114.15.2), CD11c-FITC/CD11c-PE/CD11c-APC (N418), CCR9-PE (9B1), CD103-PE/CD103-PE/Cy7 (2E7), Armenian hamster IgG isotype control-PE (HTK888), CD45-PE/Cy7 (30-F11), F4/80-APC (BM8), CCR7-APC (4B12), and CD11b-APC/Fire750 (M1/70). Live/dead cells were analysed using the Fixable Viability Dye ef450 (eBioscience) added to samples and left to incubate for 15 min at room temperature. After staining, samples were washed twice with buffer and processed using MACSQuant Analyzer (Miltenyi) and analysed with FlowJo (Tristar). Gating for ALDEFLUOR expression was determined by the negative DEAB control of each sample.

### Enteroid co-cultures with DCs

To analyse the interactions between DCs and enteroids, DCs were labelled with carboxyfluorescein succinimidyl ester (CFSE) prior to addition with enteroids derived from ROSA ^mT/mG^ mice. Enteroids were removed from Matrigel^®^ through disruption with PBS and centrifuged at 300×*g* for 5 min. Cultured DCs or those isolated from the siLP were re-suspended in Matrigel^®^ at a density of 1.1 × 10^5^/ml and added to the enteroids. The samples were cultured onto glass bottom culture dishes and incubated at 37°C in 5% v/v atmospheric CO_2_ for 20 min for Matrigel^®^ polymerization, followed by the addition of advanced phenol-red-free DMEM/F12 medium (ThermoFisher).

To analyse the conditioning of DCs in culture with enteroids, DCs were re-suspended in Matrigel^®^ at a density of 2 × 10^6^/ml and added to enteroids derived from C57B1/6J mice. The samples were aliquoted onto coverslips within a well plate, incubated at 37°C in 5% v/v atmospheric CO_2_ for 20 min for Matrigel^®^ polymerization, with subsequent addition of enteroid medium. Cultures were incubated for 24 h and resuspended in PBS by vigorous pipetting for flow cytometry analysis.

Circularity measurements of enteroids were determined by outlining the enteroids in Image J software to obtain perimeter (*P*) and area (*A*) measurements used to acquire a circularity value with the equation 4π*A/P*^2^.

Supernatant cytokine levels were analysed using the LegendPlex Mouse Inflammation panel (Biolegend) in accordance with the manufacturer’s instructions. Data were acquired on a MacsQuant analyser (Miltenyi) and analysed using FlowJo (Tristar).

### Two-photon microscopy and image analysis

Time-lapse, z-stack images of co-cultures were acquired over a 50-min period at 2-min intervals using Zen Black software on a Zeiss LSM 880 MP microscope (Zeiss) and a two-photon laser set to 920 nm (Coherent). Emission light was separated with 490 nm or 555 nm dichroics with bandpass filters 525/25 nm and 590/20 nm used to minimize spectral overlap.

Images were processed using Imaris x64 v9.0.1 (BitPlane AG; Zurich, Switzerland). Morphology of DCs was assessed using the surface functions and motility using the ‘spots’ function. All automated surface coverage and cell tracking was manually checked for accuracy.

### Statistical analysis

Results are expressed as means ± SEM. Two-way ANOVA with Tukey’s post-comparison test was used to determine statistical significance among multiple groups, and *t*-test was used to determine statistical significance between two groups. *P < 0.05, **P < 0.01, ***P < 0.001, ****P < 0.0001.

## Results

### Sequential exposure of bone marrow progenitors to Flt3L and GM-CSF/RA results in the generation of DCs with gut-like characteristics

While enteroid models have been useful in understanding intestinal development and homeostasis, the lack of an immune cell component is a limiting factor. In order to better understand how cross-talk between intestinal epithelial cells and cDCs contributes to intestinal immune homeostasis, we set out to develop an enteroid–cDC co-culture model.

**Figure 1:**
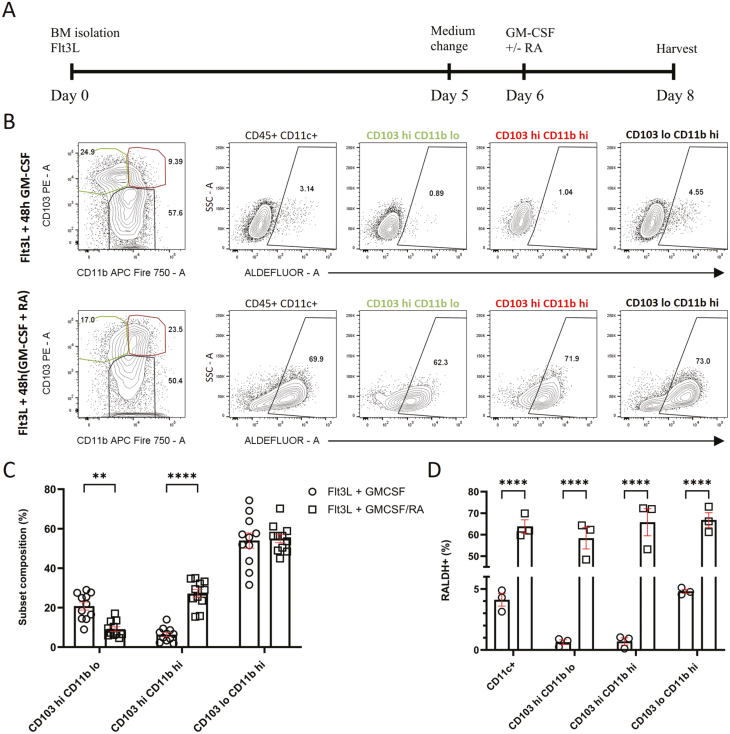
Sequential exposure of bone marrow progenitors to Flt3L and GM-CSF/RA results in the generation of DCs with gut-like characteristics. (**A**) Bone marrow cells were cultured for 8 days in 200 ng/ml Flt3L, with 20 ng/ml GM-CSF or 20 ng/ml GM-CSF plus 1 µM RA added for the last 48 h of culture. Cells were harvested and enriched for CD11c^+^ cells by MACS on day 8 of culture. (**B**) Flow cytometric analysis of RALDH activity in BMDC populations. Gated CD45^+^CD11c^+^ cells were sub-gated into CD103^hi^CD11b^lo^, CD103^hi^CD11b^hi^, and CD103^lo^CD11b^hi^ populations, and positive staining with ALDEFLUOR determined using a DEAB control. Numbers indicate the percentage of cells in the gate. (**C**) The graph depicts the percentage of CD45^+^CD11c^+^ cells in the indicated gate. Results are pooled data from 11 independent experiments, with mean ± SEM shown. Two-way ANOVA with Tukey’s multiple comparisons test. (**D**) The proportion of cells within each subset (defined by CD11b and CD103 expression) expressing ALDEFLUOR staining is shown. Data are pooled from three independent experiments, with mean ± SEM shown. Two-way ANOVA with Tukey’s multiple comparisons test. **P < 0.005, ****P < 0.0001.

cDCs harvested from the intestinal LP are too low for high-throughput experiments, and the use of enzymes such as collagenase during the isolation process may affect cDC phenotype and function. Current methods for generating gut-like cDCs from bone marrow progenitors provide good cell yields but can generate heterogeneous mixtures of DCs and macrophages, or require the pre-treatment of mice with Flt3L and sorting for pre-mucosal DCs [7, 24, 31–34]. Therefore, a reliable and straightforward method to generate pure DCs with an intestinal phenotype from the bone marrow of untreated mice would be a valuable resource. The development of intestinal cDCs from haematopoietic stem cells requires exposure to Flt3L, GM-CSF, and RA. Inclusion of all three factors from day 0 to day 8 of bone marrow culture resulted in a heterogenous population of DCs and macrophages, similar to GM-CSF-only culture protocols (Supplementary Fig. 1; [31]). Flt3L is predominantly expressed in the bone marrow, while RA and GM-CSF are also produced by stromal cells and innate lymphoid cells in the small intestine [11–14]. This suggests that the development of small intestinal cDCs is controlled by sequential exposure to these factors (Fig. 1A). Indeed, inclusion of GM-CSF only on days 6–8 of Flt3L bone marrow culture resulted in the generation of CD11c^+^ DCs, but not contaminating F4/80^+^ macrophages (Supplementary Fig. 1). However, as expected, the resultant DCs lacked RALDH activity and the prominent CD103^hi^CD11b^hi^ cDC2 population found in the small intestine, instead consisting primarily of CD103^hi^CD11b^lo^ cDC1 and CD103^lo^CD11b^hi^ cDC2-like populations (Fig. 1B–D). The of RA for the last 48 h of culture resulted in an expansion of a CD103^hi^CD11b^hi^ cDC2-like population, and the induction of RALDH activity across all DC sub-populations (Fig. 1B–D). Flt3L bone marrow-derived DCs (BMDCs) cultured with GM-CSF and RA for the final 48 h phenotypically resembled small intestinal cDCs and will be referred to as bone marrow-derived gut-like cDCs (BMgDCs). Flt3L BMDCs cultured with GM-CSF for the final 48 h will be used as a comparator and referred to as BMDCs.

### CD103^hi^CD11b^hi^ DCs generated in the presence of GM-CSF/RA express a cDC2 phenotype

We have established a means of generating gut-like cDCs from bone marrow that can be characterized by their expression of CD103 and CD11b, and by RALDH activity. However, we wanted to confirm that the CD103^hi^CD11b^hi^ cDC2-like population expanded in the presence of RA was representative of its *in vivo* counterpart and expressed prototypic cDC2 markers.

**Figure 2:**
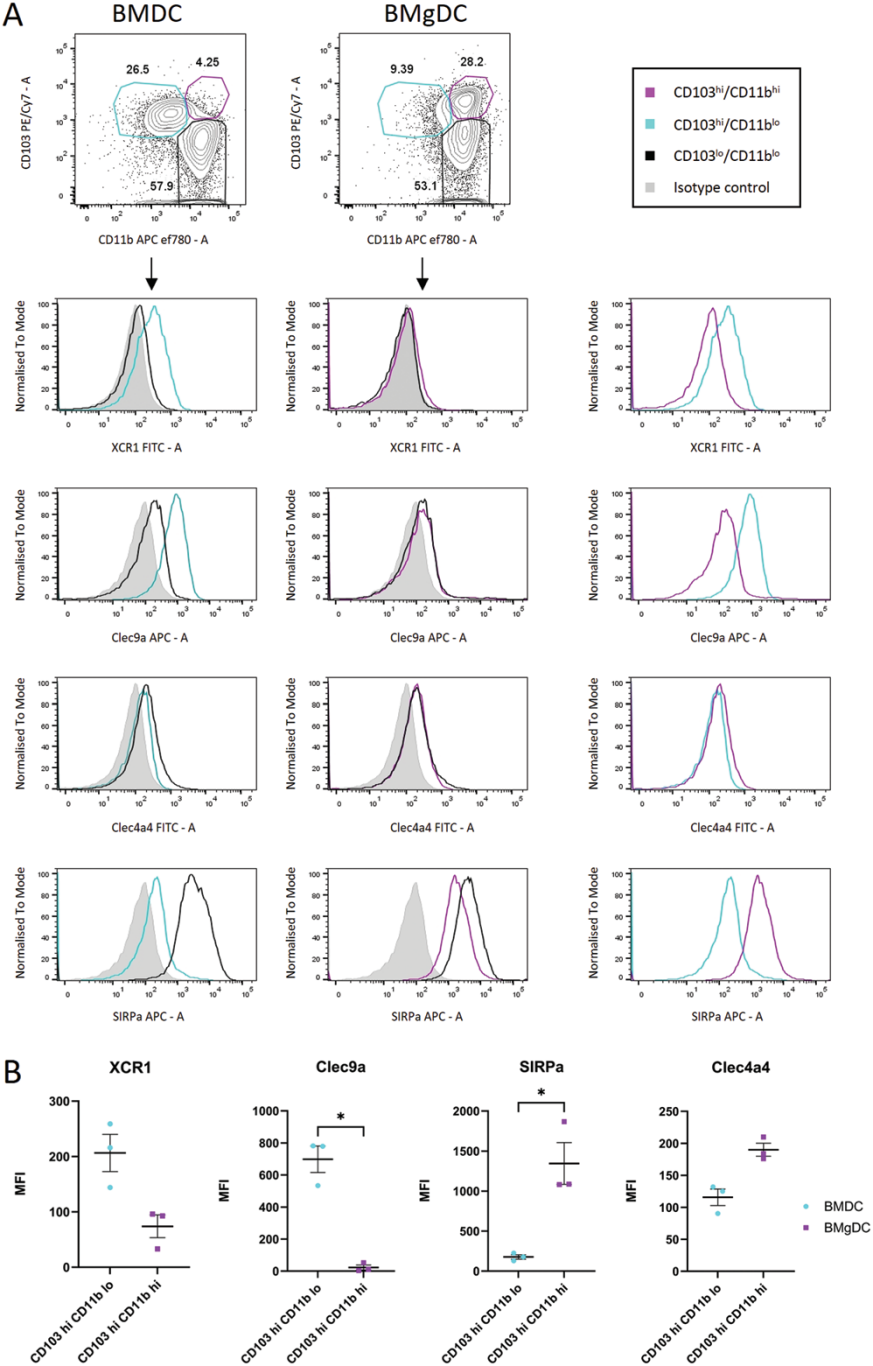
CD103^hi^CD11b^hi^ DCs generated in the presence of GM-CSF/RA express a cDC2 phenotype BMDC (Flt3L + GM-CSF) and BMgDC (Flt3L + GM-CSF/RA) cultures were analysed by flow cytometry for cDC1 and cDC2 markers. (**A,B**) Gated CD45^+^CD11c^+^ cells were sub-gated into CD103^hi^CD11b^lo^ (cyan), CD103^hi^CD11b^hi^ (purple), and CD103^lo^CD11b^hi^ (black) populations, and expression of XCR1, Clec9a, Clec4a4, and SIRPα determined by median fluorescent intensity. (**A**) Shaded histograms depict staining with an isotype control. The right panel compares CD103^hi^CD11b^lo^ (cyan) DC from BMDC cultures with CD103^hi^CD11b^hi^ (purple) DC from BMgDC cultures. (**B**) Data are pooled from three independent experiments, and the graph shows mean ± SEM. Paired *t*-test, *P < 0.05.

cDC1 and cDC2 populations can be distinguished by their expression of specific markers such as XCR1 and Clec9a in cDC1s, and SIRPα and Clec4a4 in cDC2s [35–37]. When we compared the CD103^hi^CD11b^hi^ cDC2-like population in BMgDC cultures to the CD103^hi^CD11b^lo^ cDC1-like population in BMDC cultures, we found higher expression of the cDC2 marker, SIRPα, in CD103^hi^CD11b^hi^ BMgDC, and higher expression of cDC1 markers XCR1 and Clec9a on CD103^hi^CD11b^lo^ BMDC (Fig. 2A–B). We therefore consider the CD103^hi^CD11b^hi^ BMgDC to be representative of small intestinal cDC2. Co-culture of the BMgDC population with enteroids is therefore likely to be beneficial in deciphering the immune-epithelial signalling that contributes to intestinal immune homeostasis.

### Co-cultured BMgDCs interact with the intestinal epithelium

The addition of intestinal DCs to enteroid cultures is an important step in increasing the power of enteroid models for studying intestinal homeostasis and disease. Intravital live imaging has shown that small intestinal mononuclear cells physically interact with the intestinal epithelium by crawling along the basal epithelial surface, penetrating the epithelial cell layer, and extending TEDs: important characteristics for the transfer of antigens [28, 38]. To investigate if these characteristics were present in our *in vitro* co-culture, enteroids with a fluorescent membrane label were generated from Rosa-mT/mG mice, and DCs were stained with CFSE. BMgDC, BMDC or freshly isolated siLP DC were mixed with enteroids during routine passage, before replating in Matrigel, and multiphoton live imaging. DCs were isolated from the siLP through positive selection for CD11c, with the predominant subset being cDC2 (Supplementary Fig. 2). This population is therefore a useful ‘natural’ comparator for the BMgDC population.

**Figure 3:**
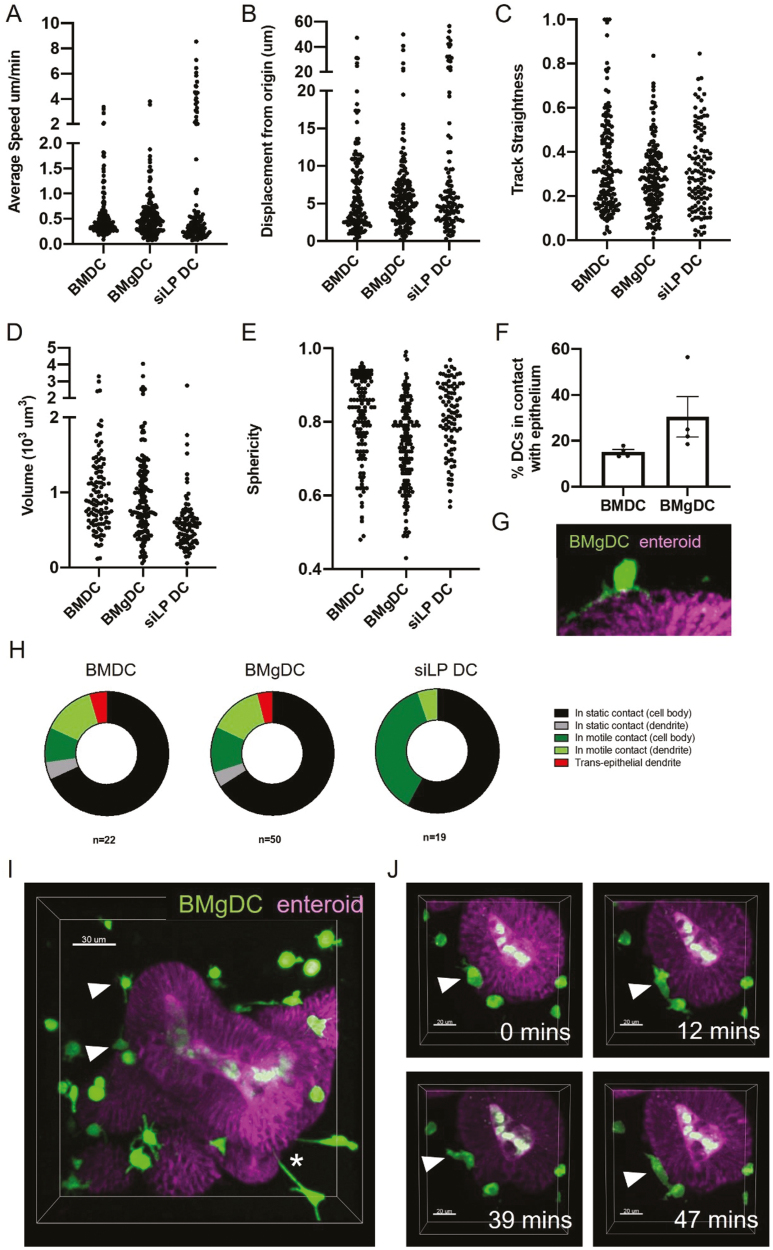
Co-cultured BMgDCs interact with the intestinal epithelium and extend TEDs BMDCs, BMgDCs, and siLP DCs were labelled with CFSE (green) and cultured with enteroids derived from ROSA^mT/mG^ mice (purple). Z-stack images were obtained at 2-min intervals over a 50-min period using a microscope equipped with a two-photon laser. Images were analysed using Imaris software to provide motility and morphology measurements of DCs, including average speed (**A**), displacement from the point of origin (**B**), track straightness (**C**), volume (**D**), and sphericity (**E**). In each case, data points indicate individual cells, and data are compiled from four independent experiments for BMDC and BMgDC, and three independent experiments for siLPDC. (**F**) The proportion of dendritic cells in direct contact with the intestinal epithelium was determined. Each data point represents an independent experiment. The mean ± SEM of four independent experiments is shown. (**G**) A BMgDC (green) in contact with the enteroid epithelium (purple) and extending a process along the basal surface (**H**). Types of contacts between DC and enteroids were visually categorized as static (immotile) contacts between the enteroid and dendritic cell body, static contacts between the enteroid and a dendrite, motile contacts between the enteroid and dendritic cell body, motile contacts between the enteroid and a dendrite, and trans-epithelial projections. Data are compiled from four independent experiments for BMDC and BMgDC, and three independent experiments for siLPDC. (**I**) 3D image of BMgDCs in co-culture with enteroids showing DCs (green) in contact with the enteroid epithelium (purple). Contacts between the dendritic cell body and enteroid are indicated with white arrows. Contacts between a dendrite and the enteroid are indicated with a white asterisk. (**J**). Time-lapse images of a BMgDC (green) crawling along the surface of an enteroid (purple).

BMgDCs, BMDCs, and siLP DCs all migrated with average speeds of <1 µm/min, though rare highly motile cells were observed in each case (Fig. 3A). Mean displacement and track straightness (an indication of directionality) were comparable in all DC types assessed, indicating that exposure to RA did not significantly alter DC migration (Fig. 3B and C). DC morphology (volume and sphericity) was comparable between BMgDC and BMDCs, while siLP DCs were smaller and more spherical than BMgDC, perhaps reflecting the lengthy isolation process (Fig. 3D and E).

DCs from all sources were seen to interact with the enteroid surface, with the proportion of BMgDCs in contact with enteroids greater than those from BMDC cultures, although not significantly so (Fig. 3F and G). Interactions between DCs and the enteroid surface were classified into four types: the DC was non-motile with the cell body in close contact with the epithelium, the DC was non-motile with a dendrite extended to contact the epithelium, the DC was motile with the cell body in close contact with the epithelium, and the DC dynamically extended a dendrite to contact the epithelium (Fig. 3H–J). BMgDC (*n* = 22) and BMDC (*n* = 50) behaved similarly, indicating that exposure to RA did not significantly alter DC behaviour (Fig. 3H). We did not observe the migration of DCs into the epithelial cell layer, nor across the epithelium into the enteroid lumen.

### DCs extend TEDs in co-culture with enteroids

A number of previous studies have shown that intestinal mononuclear phagocytes extend TEDs across the intestinal epithelium to sample antigens [27, 28, 39–41]. Often, these were CX_3_CR1^+^ macrophages that extended dendrites in response to an inflammatory stimulus, but a single study showed that CD103^+^ cDCs migrating in the epithelial layer could also extend TED to sample luminal bacteria [28]. How this sampling behaviour is regulated remains poorly understood.

**Figure 4:**
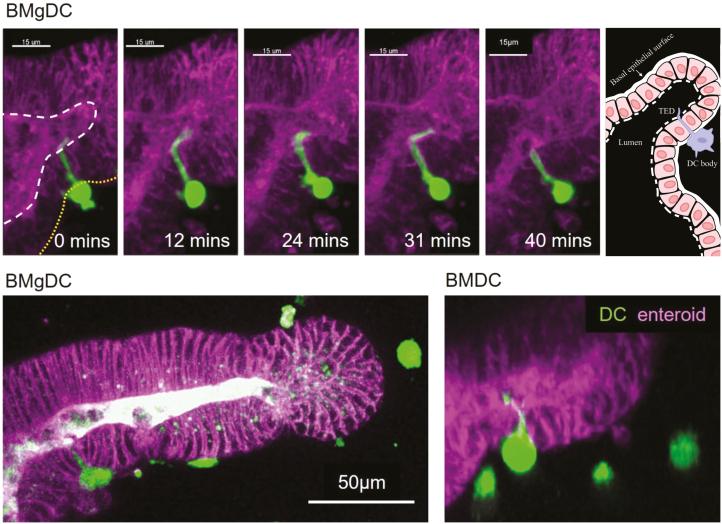
cDCs sample the enteroid lumen by extending transepithelial dendrites (TEDs). BMDCs and BMgDCs were labelled with (green) and cultured with enteroids derived from ROSA^mT/mG^ mice (purple). Z-stack images were obtained at 2-min intervals over a 50-min period. The top panel shows a cross section of a BMgDC attached to the basal surface (yellow dashed line) of an enteroid, extending a TED into lumen. The apical surface of the epithelium is indicated by a white dashed line. Scale bar: 15 µm. A cartoon schematic of the interaction is shown in the far-right segment. The bottom panel shows examples of a BMgDC and BMDC extending TED. Scale bar: 50 µm. Whole images can be viewed as Supplementary Movie M1.

Our co-culture model offers the potential to dissect the precise conditions leading to the generation of TED in different cDC populations. We observed extension of TED when both BMDC and BMgDC were co-cultured with enteroids (Fig. 4 and Supplementary Movie M1). DCs attached to the basal surface of enteroids and extended dendrites between epithelial cells into the lumen. The luminal portion of the dendrite was motile and suggestive of sampling behaviour, although no overt stimuli were provided (Supplementary Movie M1). Our results suggest that sampling behaviour is a characteristic of cDC and opens up opportunities to better understand its regulation and function.

### Co-culture of BMgDC with enteroids does not lead to epithelial damage

**Figure 5:**
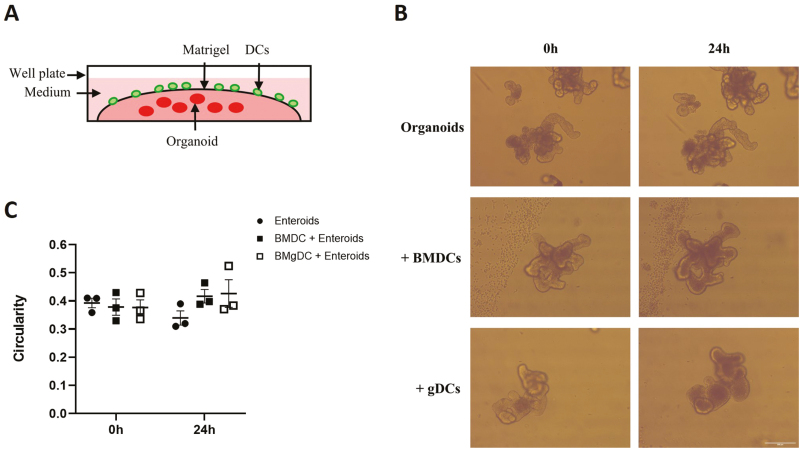
Co-culture with DCs did not increase the circularity of enteroids. (**A**) BMDCs and BMgDCs were added to established enteroid cultures at 2 × 10^6^ cells/ml. (**B**) Brightfield images of enteroids were taken at 0 h and 24 h and (**C**) used to calculate circularity (defined by combining perimeter (*P*) and area (*A*) measurements in the equation 4π*A/P*^2^) of enteroids using ImageJ software. Each data point represents the mean of an independent experiment. For each independent experiment, duplicate cultures were performed, with three images obtained per sample. The graph depicts mean ± SEM. Two-way ANOVA with Tukey’s multiple comparisons test. Scale bar = 200 µm.

Enteroid ‘rounding’ has been shown to correlate with increased epithelial cell apoptosis and shedding, and is induced following exposure to inflammatory cytokines or LPS-activated BMDC [4]. Since we primarily wanted a co-culture model that would replicate steady-state conditions, we wanted to confirm that co-culture of enteroids with BMgDC would not result in epithelial cell death and enteroid rounding. We did not see any increase in enteroid circularity (defined by combining perimeter [*P*] and area [*A*] measurements in the equation 4π*A/P*^2^) after 24 h of co-culture with BMDC or BMgDC, indicating that both populations exist in a non-inflammatory state, and allowing for immune-epithelial cross-talk to be investigated for a minimum period of 24 h (Fig. 5).

### RALDH activity in cDC is decreased following co-culture with enteroids

**Figure 6:**
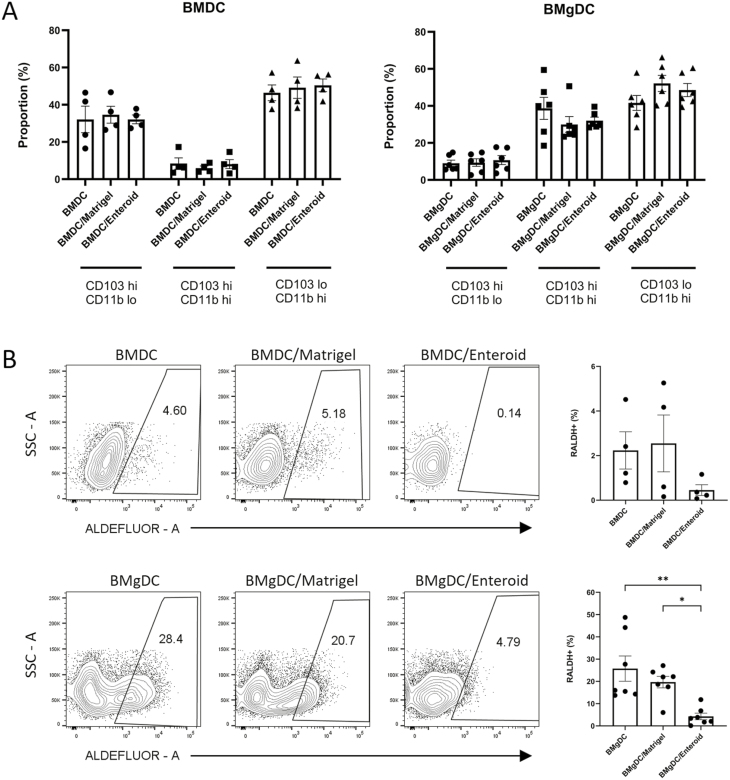
Enteroids suppress ALDH activity in DCs. BMDCs and BMgDCs were cultured alone, in Matrigel, or in Matrigel with enteroids derived from C57Bl/6 mice for 24 h. Subset composition (**A**) and RALDH activity (ALDEFLUOR) (**B**) were analysed by flow cytometry. (**A**) CD45^+^CD11c^+^ cells were sub-gated into CD103^+^CD11b^-^, CD103^+^CD11b^+^, and CD103^-^CD11b^+^ populations. The graph depicts proportions of each DC subset either cultured alone, in Matrigel, or with enteroids. Each data point represents an individual experiment (duplicate conditions). Results are pooled from four to six independent experiments, with mean ± SEM shown. (**B**) The percentage of CD45^+^CD11c^+^ cells staining for ALDEFLUOR is shown (gates determined using DEAB control). Each data point represents an individual experiment (duplicate conditions). Results are pooled from four (BMDC) or seven (BMgDC) independent experiments, with mean ± SEM shown. *P < 0.05, **P < 0.005. Two-way ANOVA with Tukey’s comparison test.

The intestinal epithelium is believed to provide signals that contribute to the development and functional imprinting of the CD103^+^CD11b^+^ cDC2 population. This includes epithelium-derived factors such as TGF-β, MUC2, cellular retinol-binding protein II (CRBPII), and RA [3, 20, 42]. We therefore expected that co-culture of our BMDC population with enteroids would lead to an accumulation of CD103^+^CD11b^+^ cDC2, and induction of RALDH activity, analogous to what we observed following the addition of RA to bone marrow cultures. In fact, we observed no difference in DC subset composition, nor an increase in RALDH activity, following co-culture of BMDC with enteroids (Fig. 6A). Similarly, no difference in DC subset composition was observed following co-culture of BMgDC with enteroids (Fig. 6A). Unexpectedly, we found that RALDH activity in BMgDCs was significantly suppressed following co-culture with enteroids (Fig. 6B). Culturing BMgDC in matrigel and medium alone did not result in a loss of RALDH activity, suggesting that the enteroid epithelium may be producing a factor that inhibits RALDH activity in DCs (Fig. 6B).

### Continued provision of GM-CSF and RA during co-culture opposes negative signals from the enteroid epithelium and maintains RALDH activity in DCs

Several different environmental cues have been shown to promote RALDH activity in DCs, and we reasoned that additional, non-epithelial, factors may be required to maintain RALDH activity in co-culture. These include the microbial metabolite butyrate, the cytokine IL-22, a source of retinol for epithelial cells to metabolize to RA, and TLR ligands [22, 43–46]. However, in preliminary experiments, the addition of IL-22, butyrate, retinol or CpG to co-cultures could not maintain RALDH activity in BMgDCs (Supplementary Fig. 3). Failure of retinol to restore RALDH activity in DCs argues against simple consumption of RA by enteroids as a mechanism for impaired RALDH activity in co-cultured DCs. Rather, we believe that negative signals from the intestinal epithelium oppose RALDH activity in DCs.

**Figure 7:**
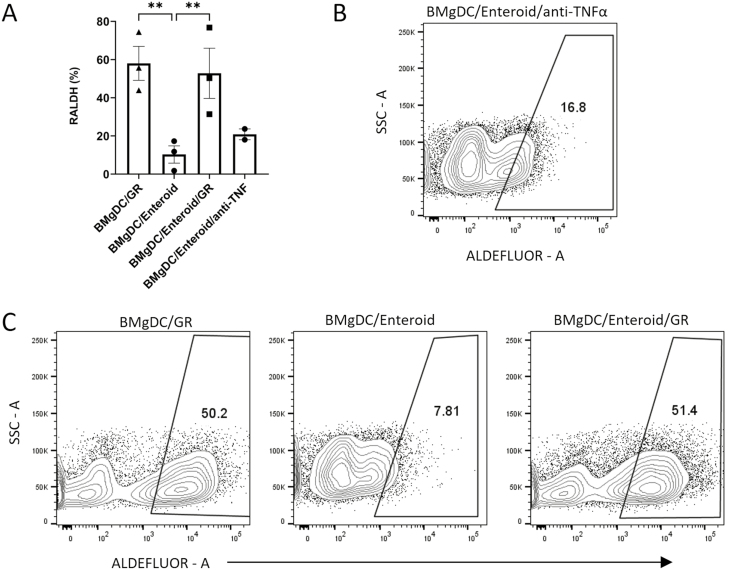
Supplementing co-cultures with GM-CSF and RA restores ALDH expression in BMgDCs. BMgDCs were cultured with enteroids for 24 h in the presence or absence of GM-CSF and RA (GR) or with anti-TNF-α antibody. RALDH activity in DCs at the end of the culture period was determined by flow cytometry. (**A**) The percentage of CD11c^+^ cells staining for ALDEFLUOR is shown. (**B**) ALDEFLUOR staining for the anti-TNF-α condition. (**C**) Representative flow plots of BMgDC cultures with enteroids and GR. Each data point represents an individual experiment. Results are pooled from two to three independent experiments, with mean ± SEM shown. *P < 0.05. Two-way ANOVA with Tukey’s comparison test.

We next investigated the possible identity of this negative signal. TNF-α has been shown to reduce RALDH activity in human DCs [47]. However, the addition of an anti-TNFα antibody to BMgDC and enteroid co-cultures failed to restore ALDH expression (Fig. 7A and B).

Since GM-CSF and RA are produced in the small intestine by stromal cells and the epithelium, respectively, we investigated if the continued presence of these factors during co-culture would support RALDH activity in DCs. The addition of RA and GM-CSF to enteroid-BMgDC restored RALDH activity, suggesting that their continued presence is required to oppose negative signals from the enteroid epithelium (Fig. 7A and C).

## Discussion

The addition of an immune component to enteroid cultures is an important step in increasing the power of enteroid models for studying intestinal homeostasis and disease. Here, we have described a simple method for the generation of ‘gut-like’ CD103^+^CD11b^+^ cDC2 with high RALDH activity. These DCs can be co-cultured with enteroids, where they form dynamic interactions with epithelial cells, resulting in phenotypic modulation. Surprisingly, we found that the intestinal epithelium negatively regulated RALDH activity in DCs and that the continued presence of RA/GM-CSF was required to overcome these inhibitory signals. Our model therefore offers opportunities to better understand the specific molecular signals that pass between epithelial cells and DCs, and how they contribute to tissue homeostasis and disease.

Since their first description more than a decade ago, enteroid cultures have allowed valuable insight into the development, physiology, and pathology of the intestinal epithelium [48–52]. However, the functional properties of the intestinal epithelium are intimately associated with the extensive network of underlying immune cells, which contribute to epithelial barrier function and repair. Epithelial-derived molecules also regulate immune cell function, allowing for non-destructive regulation of the microbial communities that reside in the gut, while the microbiota regulates both immune and epithelial function. Clearly, this complex interplay is difficult to dissect in an *in vivo* setting, where variations in diet, microbial communities and stress can confound experimental data. Consequently, there is much we still do not understand about the molecular signals that pass between the epithelial and immune compartments, and how they change in disease. We consider that a reductionist *in vitro* cellular model will be an important tool in dissecting these interactions under experimentally defined conditions, and make the surprising observation that the intestinal epithelium may actually oppose RALDH activity in DCs.

Other studies have described the co-culture of immune cells with gastrointestinal organoids [2–4, 53–63]. Macrophages have been co-cultured with human enteroid-derived monolayers, resulting in enhanced barrier function, while human monocyte-derived DCs follow chemotactic cues to interact with the epithelium of gastric spheroids [2, 60]. Other studies have utilized conventional BMDC or DC isolated from the siLP to demonstrate cross-talk between mononuclear phagocytes and the organoid epithelium, primarily focussing on infected organoids or activated phagocytes [2, 4, 61]. However, the small intestine has a unique CD103^+^CD11b^+^RALDH^+^ cDC2 population that is not represented in murine GM-CSF-BMDC or human monocyte-derived DC populations, and is challenging to isolate in sufficient numbers from the siLP. The novelty of the model presented here therefore lies in the ability to study steady-state interactions between DCs and the epithelium, while using an easily accessible population of DCs representative of those found in the intestine. Further comparison of our ‘gut-like’ DCs with their *in vivo* counterparts will be required to validate the physiological relevance of our model system. At a minimum, this would involve expansion of the flow cytometry panel, RNA-seq comparison with DC isolated from the siLP, and confirmation of the ability of ‘gut-like’ DCs to induce Foxp3 expression and regulatory function in naive T cells. This model can be utilized to define how epithelial and cDCs communicate in the intestine under a variety of different physiological conditions, including exposure to different nutrients, natural products, components of the microbiota, or pathogens [30, 48].

Our co-culture system allows for simple real-time visualization of interactions between the small intestinal epithelium and cDCs, circumventing challenging *in vivo* protocols. While numerous studies have performed dynamic multiphoton imaging of the small intestine, this technique requires anaesthesia and surgical exposure of the intestine, or the maintenance of explanted tissue in oxygenated media [27, 28, 38, 39, 64–66]. In both cases, the window of time for imaging is short before damage to the epithelium occurs, or peristaltic movement hinders image acquisition. Imaging from the serosal surface is less damaging, but imaging depth is rarely sufficient to capture events in the villous epithelium at high resolution. Using our co-culture model, we were able to capture the extension of DC TEDs across the epithelial cell layer and into the organoid lumen. This was observed in the absence of RA/GM-CSF; therefore, the influence of these factors on TED formation is yet to be investigated. This activity is typically thought to represent antigen sampling behaviour which may be important for both maintaining an equilibrium with the microbiota and for the generation of protective immune responses to pathogens. It is therefore important to understand which cell types engage in this behaviour and how it is regulated.

Extension of TEDs has most often been associated with CX_3_CR1^+^ macrophages [27, 39–41, 67]. In this scenario, the formation of TEDs is mediated by CX_3_CR1^+^ and is enhanced in the presence of TLR ligands or pathogenic bacteria. While some reports suggest that CD103^+^ cDCs instead acquire antigen from CX_3_CR1^+^ macrophages or via goblet cell-associated antigen passages (GAPs), CD103^+^ cDC2 have also been found to colonize and patrol the small intestinal epithelium in response to RA [3, 28]. Once in the epithelium, they undergo transcriptional reprogramming in response to epithelial signals and extend TEDs in response to bacterial challenge [3, 28]. However, the molecular cues that stimulate this behaviour in cDC2 remain ill-defined. Because our model allows DC–epithelium interactions to be directly visualized over longer time periods, and to be manipulated in real time, we have the opportunity to precisely define how cDC TEDs are regulated under different conditions.

An unexpected finding was that the intestinal epithelium negatively regulated RALDH activity in DCs, and that the continued presence of RA/GM-CSF was required to overcome these inhibitory signals. RA and GM-CSF are known to synergistically induce RALDH activity in DCs and to contribute to the development and maintenance of the CD103^+^CD11b^+^ cDC2 population in the intestine [68]. In the intestine, GM-CSF is produced by CD31^−^Epcam^−^Pdpn^hi^ stromal cells or ILC3, while RA is contributed by epithelial cells or CD31^−^Epcam^−^Pdpn^hi^ stromal cells [11–14, 42]. These stromal cell populations are not present in our co-culture system, and it is possible that the enteroid epithelium has lost the capacity to produce RA from retinol. However, CD103^+^CD11b^+^ cDC2 with high RALDH activity (BMgDC) were maintained following 24 h of culture in Matrigel alone, and it was only when enteroids were added that RALDH activity was inhibited. These data suggest that it is not the simple absence of RA/GM-CSF that causes the loss of RALDH activity in BMgDC. We therefore propose that RA/GM-CSF are not just required to induce an intestinal cDC2 phenotype, but also to maintain it in the face of inhibitory signals from the epithelium.

In the present study, we were unable to identify the epithelial signals that inhibited RALDH activity in DCs. Vitamin A metabolism in the intestine can be negatively regulated by the microbiota [69], PGE2 [23], or inhibition of Wnt signalling [70]. In the steady state, the intestinal epithelium can produce PGE2 and antagonists of Wnt signalling [71, 72]. It is also possible that enteroids may act as a sink for activators of Wnt signalling, otherwise required to maintain RALDH activity in DCs. An alternative explanation may be that apparently normal enteroid cultures are experiencing cellular stress, and producing inflammatory signals, which would impact upon DC phenotype [73]. However, we were unable to detect any of a panel of inflammatory cytokines in enteroid supernatants (data not shown).

In the steady state, production of RA by intestinal DCs drives the differentiation of Foxp3^+^ T cells, which suppress harmful response to the microbiota and prevent intestinal inflammation. This raises the question of whether infection or inflammation disrupts RA/GM-CSF signalling to DCs, allowing epithelial signals to inhibit RALDH activity in DCs, releasing them to stimulate Th1 responses. Our model provides a useful steppingstone in understanding the precise molecular signals exchanged between the intestinal epithelium cells and DCs, and how these may be manipulated therapeutically to resolve inflammation.

## Supplementary Material

kyad018_suppl_Supplementary_Figure_S1

kyad018_suppl_Supplementary_Figure_S2

kyad018_suppl_Supplementary_Figure_S3

kyad018_suppl_Supplementary_Movie

## Data Availability

The data that support the findings of this study are available from the corresponding authors J.C. and L.J., upon reasonable request.

## Abbreviations:

BMgDCs: bone marrow-derived gut-like cDCs;
BMDCs: bone marrow-derived DCs;
cDCs: conventional dendritic cells;
CFSE: carboxyfluorescein succinimidyl ester;
DCs: dendritic cells();
EDTA: ethylenediamine tetraacetic acid;
GM-CSF: granulocyte macrophage colony-stimulating factor;
HBSS: Hanks’ balanced salt solution;
LP: lamina propria;
RA: all-*trans*-retinoic acid;
RALDH: retinaldehyde dehydrogenase;
siLP: small intestinal lamina propria;
TED: *trans*-epithelial dendrites.

## Permission to reproduce

The present article does not include any reproduced material from other sources. All content presented is original and does not infringe upon any copyrights or intellectual property rights of others.
