# Supplementary figures and images for: A novel in vitro model of the small intestinal epithelium in co-culture with ‘gut-like’ dendritic cells
Source: Discov Immunol. 2023 Oct 7;2(1):kyad018. doi: 10.1093/discim/kyad018 (PMC10917230; doi:10.1093/discim/kyad018)

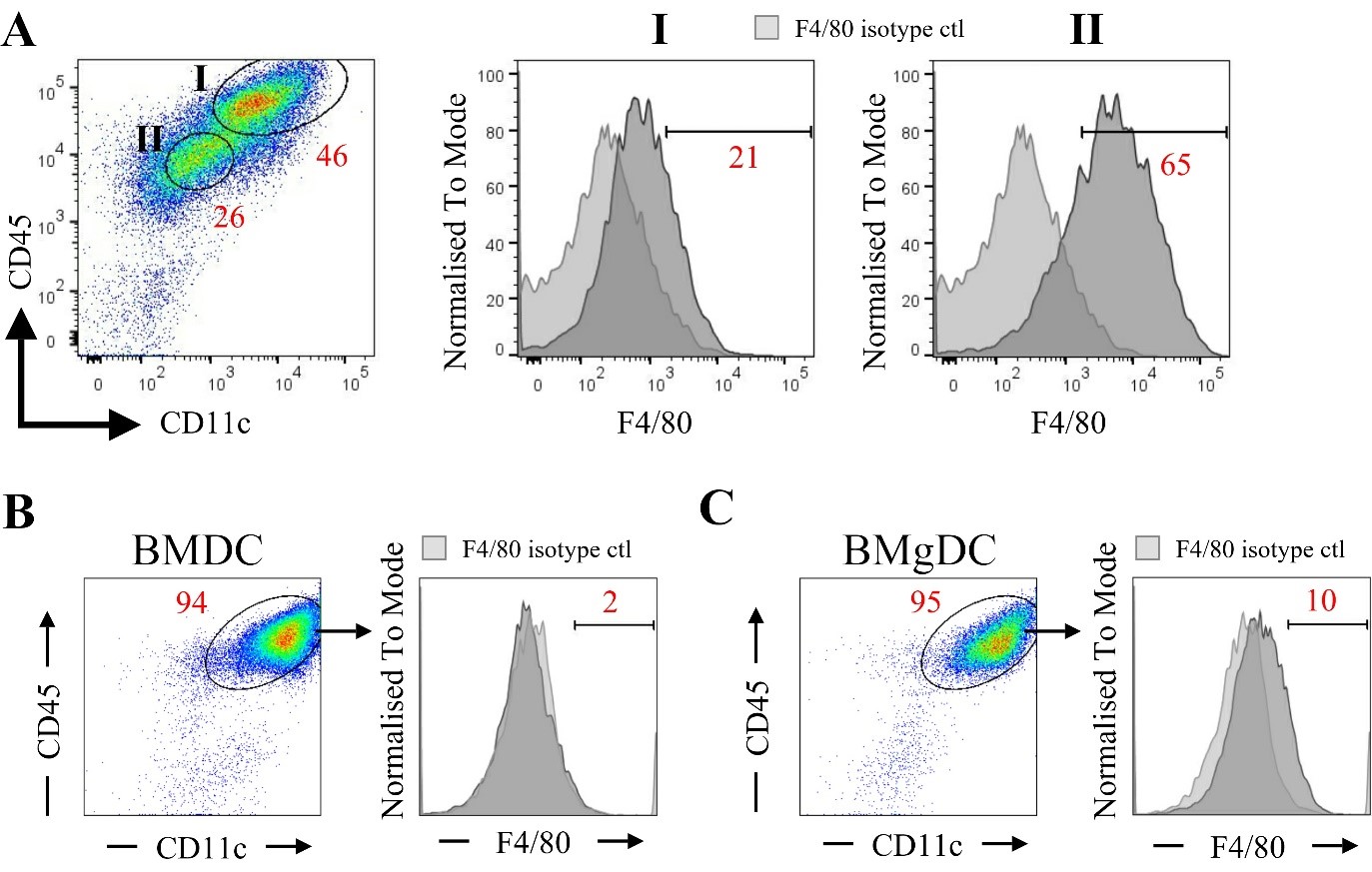

Supplement: kyad018_suppl_Supplementary_Figure_S1 [file kyad018_suppl_Supplementary_Figure_S1.jpg]

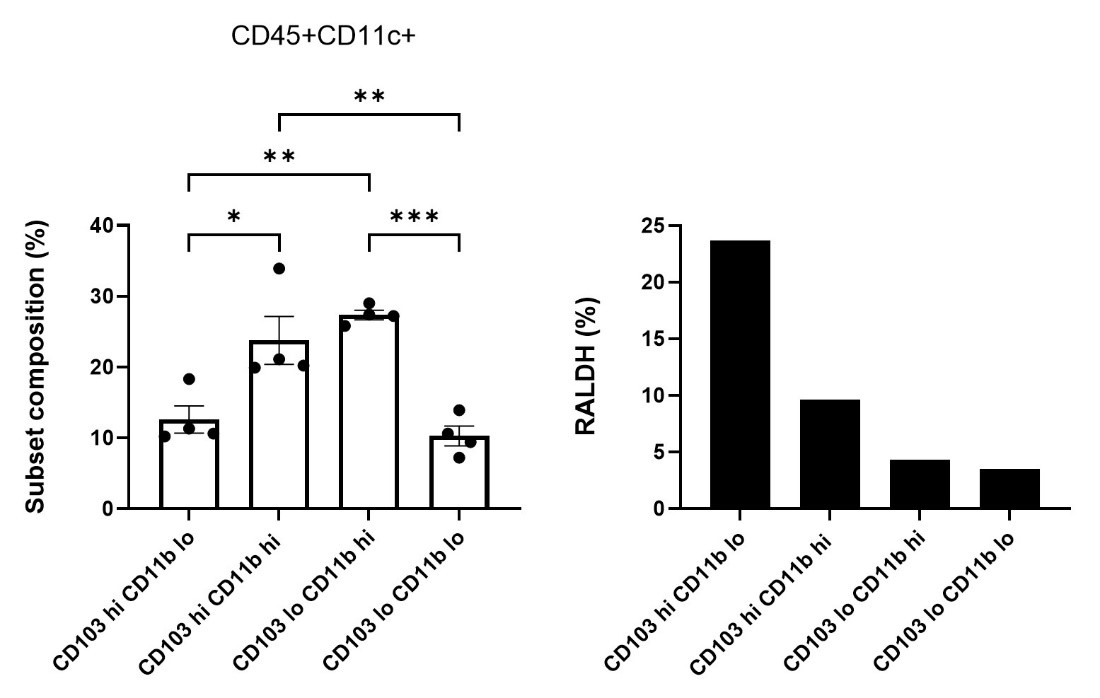

Supplement: kyad018_suppl_Supplementary_Figure_S2 [file kyad018_suppl_Supplementary_Figure_S2.jpg]

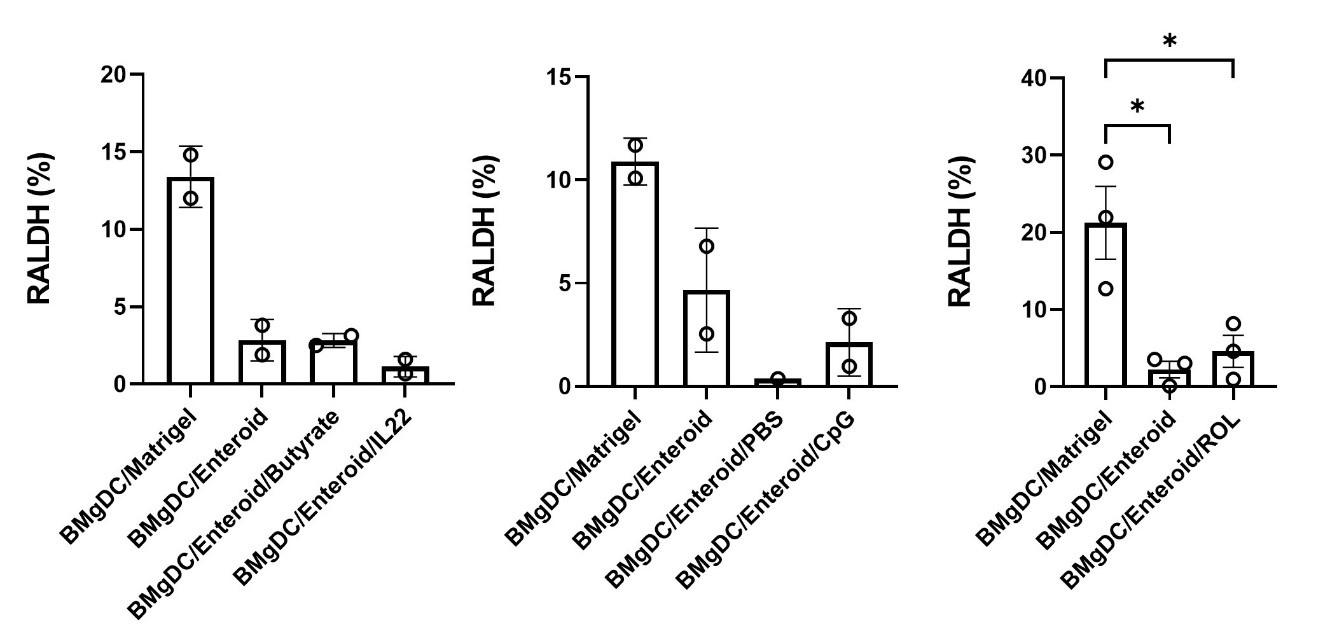

Supplement: kyad018_suppl_Supplementary_Figure_S3 [file kyad018_suppl_Supplementary_Figure_S3.jpg]
